# Functional Imaging of Pheochromocytoma with ^68^Ga-DOTATOC and ^68^C-HED in a Genetically Defined Rat Model of Multiple Endocrine Neoplasia

**DOI:** 10.1155/2011/175352

**Published:** 2011-06-08

**Authors:** Matthias Miederer, Sara Molatore, Ilaria Marinoni, Aurel Perren, Christine Spitzweg, Sybille Reder, Hans-Jürgen Wester, Andreas K. Buck, Markus Schwaiger, Natalia S. Pellegata

**Affiliations:** ^1^Department of Nuclear Medicine, Johannes Gutenberg University of Mainz, Langenbeck Strasse 1, 55131 Mainz, Germany; ^2^Institute of Pathology, Helmholtz Zentrum München, Ingolstaedter Landstrasse 1, 85764 Neuherberg, Germany; ^3^Institute of Pathology, University of Bern, Murtenstrasse 31, 3010 Bern, Switzerland; ^4^Department of Medicine, Klinikum Großhadern, Ludwig Maximilians University (LMU), Marchioninistrasse 15, 81377 Munich, Germany; ^5^Department of Nuclear Medicine, Technical University Munich, Ismaninger Strasse 22, 81675 Munich, Germany

## Abstract

Rats affected by the MENX multitumor syndrome develop pheochromocytoma (100%). Pheochromocytomas are uncommon tumors and animal models are scarce, hence the interest in MENX rats to identify and preclinically evaluate novel targeted therapies. A prerequisite for such studies is a sensitive and noninvasive detection of MENXassociated pheochromocytoma. We performed positron emission tomography (PET) to determine whether rat pheochromocytomas are detected by tracers used in clinical practice, such as 68Ga-DOTATOC (somatostatin analogue) or ^11^C-Hydroxyephedrine (HED), a norepinephrine analogue. We analyzed four affected and three unaffected rats. The PET scan findings were correlated to histopathology and immunophenotype of the tumors, their proliferative index, and the expression of genes coding for somatostatin receptors or the norepinephrine transporter. We observed that mean 68Ga-DOTATOC standard uptake value (SUV) in adrenals of affected animals was 23.3 ± 3.9, significantly higher than in control rats (15.4 ± 7.9; *P* = .03). The increase in mean tumor-to-liver ratio of ^11^C-HED in the MENX-affected animals (1.6 ± 0.5) compared to controls (0.7 ± 0.1) was even more significant (*P* = .0016). In a unique animal model, functional imaging depicting two pathways important in pheochromocytoma biology discriminated affected animals from controls, thus providing the basis for future preclinical work with MENX rats.

## 1. Introduction

Pheochromocytomas are rare neuroendocrine tumors that arise from adrenal chromaffin cells. Secretion of catecholamines by pheochromocytomas may result in clinical hypertension which is potentially life-threatening to patients. Up to 10% of pheochromocytomas will undergo malignant transformation with metastatic spread mainly to the bones and liver [1, 2]. Once metastasized, there is no curative treatment for this disease. 

Diagnosis of pheochromocytoma usually involves, at the biochemical level, the measurement of plasma or urine content of catecholamines and their metabolites, and at the macroscopic level morphological appearance with radiologic imaging, such as computed tomography and magnetic resonance tomography. More recently, functional imaging using for example, ligands specific for catecholamine uptake, synthesis/secretion pathways, or endocrine cell surface receptors has been applied for pheochromocytoma detection, in addition to classical morphological imaging and biochemical tests, to increase sensitivity and accuracy [3]. Since clinical, biochemical, and anatomic appearance may not with certainty distinguish between malignant and benign tumors, functional imaging might add critical information pre- and postoperatively improving patient management. 

The main therapeutic target for pheochromocytoma is surgical tumor elimination or reduction and control of symptoms of excessive catecholamine secretion. Currently, the best adjunctive therapy in malignant and metastasized pheochromocytoma is treatment with radiopharmaceuticals such as ^131^I-metaiodobenzylguanidine (^131^I-MIBG), which takes advantage of the norepinephrine transport system of adrenal chromaffin cells [4, 5]. However, although it often achieves successful palliation, ^131^I-MIBG therapy has limited effect on tumor control and it is generally not curative [6, 7].

To develop effective anticancer treatments, it is necessary to have available suitable preclinical models to test novel agents and to monitor their effectiveness against the tumor. This is especially important for uncommon tumors, such as pheochromocytoma, where comprehensive clinical trials are often difficult to set up and carry to completion. In a spontaneous rat model of multiple endocrine neoplasia, named MENX, pheochromocytoma develops in the affected animals with complete penetrance (100%). A clear progression from adrenal medullary hyperplasia to adenoma is evident [8]. This syndrome is inherited as a recessive trait and is caused by a germline mutation of the cell cycle regulatory gene *Cdkn1b*, encoding p27Kip1 [9].

In the current study, we sought to identify an imaging modality that would allow us to sensitively detect the adrenal tumors that occur in the MENX-affected rats. We here demonstrate that adrenal lesions can be detected by ^11^C-HED, a tracer that takes advantage of the norepinephrine transport system which is expressed in adrenal chromaffin cells. Detection of these neuroendocrine tumors is also possible with a somatostatin receptor ligand (^68^Ga-DOTATOC). Quantification of distinct functional parameters in this animal model of pheochromocytoma is a prerequisite to exploit this system for the preclinical testing of novel treatment regimens *in vivo* and for the evaluation of novel imaging modalities. Based on our findings, ^11^C-HED-PET could be used to monitor noninvasively tumor behaviour following treatment of MENX rats with antitumor drugs, allowing repeated investigations of the same animals throughout the treatment procedure. 

## 2. Materials and Methods

### 2.1. Animals

The MENX phenotype was initially identified in a Sprague Dawley (SD) rat colony and maintained as previously reported [8]. Affected rats are homozygous for a germline frameshift mutation in the *Cdkn1b* gene (p27Kip1) and are hereafter indicated as affected or mut/mut [9]. The affected rats spontaneously develop pheochromocytoma and other neuroendocrine tumors [8]. Animals were maintained in agreement with general husbandry rules approved by the Helmholtz Zentrum München. Rats were treated in accordance with the procedures approved and recommended by the provincial government (Bayerische Landesregierung). 

### 2.2. Anatomical and Histological Analysis of Rats

Four 5-months-old mut/mut rats from the breeding colony and three age-matched unaffected littermates (controls) were subjected to imaging procedures outlined below. Following imaging, all rats were euthanized with carbon dioxide in compliance with institutional requirements and subjected to complete necropsy. Body and organ weights were measured for every animal during necropsy. For histological analysis, half of the adrenal gland was formalin-fixed, paraffin-embedded, and evaluated using established criteria for neoplasia of the rat endocrine system. The other half of the adrenal gland was snap-frozen for molecular analyses. To calculate the volume of the adrenal glands, we used the ellipsoid formula, using the shortest diameter as the third dimension. 

### 2.3. Pet Imaging

The radiotracers ^68^Ga-DOTATOC and ^11^C-Hydroxyephedrine (^11^C-HED) were synthesized according to routine protocols. 

PET radiotracers were injected intravenously via tail veins (1 *μ*g/18 MBq for ^68^Ga-DOTATOC; 5 MBq for ^11^C-HED), and animals were imaged after sedation with isoflurane, which was maintained during imaging procedures. 15 min static PET images were acquired after 45 min distribution for the longer lived PET tracer ^68^Ga-DOTATOC (*T*_1/2_ = 67 min); for the shorter lived ^11^C-HED (*T*_1/2_ = 20 min) after 5 min distribution time (Siemens Inveon dedicated PET, Siemens, Erlangen), 10 min static images were acquired. Image reconstruction was performed with an OSEM2d iterative algorithm into a 128 × 128 × 159 matrix with a voxel size of 0.776 × 0.776 × 0.796 mm^3^. For ^68^Ga-DOTATOC images attenuation (10 min point source singles based), and scatter correction was applied to facilitate absolute quantification. All data were corrected for random, deadtime and decay. 

### 2.4. Image Analysis

Image analysis was done with the PMOD 2.9 software package (PMOD Technologies, Adliswil, Switzerland). Spherical volumes of interest with a 4 mm diameter were centrally placed into adrenals of the animals used for the quantification studies (^68^Ga-DOTATOC and ^11^C-HED). Mean activity for ^68^Ga-DOTATOC studies was then corrected by injected dose and weight of the animals to derive SUV values (SUV = mean activity (kBq/mL) *weight of the animals (g)/injected dose (kBq)). Semiquantitative analysis for ^11^C-HED-studies was done by adrenal to liver ratio using mean activity from an 8 mm VOI centrally placed in the liver. 

### 2.5. RNA Extraction and Real-Time Quantitative RT-PCR

For total RNA extraction from rat snap-frozen tissues, serial 40 *μ*m tissue sections were subjected to manual macrodissection under the microscope to obtain adrenal medullary cells. Macrodissected cells were resuspended in Trizol (Invitrogen, Darmstadt, Germany) and processed following the manufacturer's instructions. One microgram of total mRNA was used to synthesize first-strand cDNA using random hexamers. Quantitative RT-PCR was done with TaqMan Assay-on-Demand primers and probes for somatostatin receptors 2 (*Sstr2*), 3 (*sstr3*), and 5 (*sstr5*), norepinephrine transporter (*Slc6a2*), and for ß2-microglobulin (*B2M*) as internal control (Applied Biosystems). The relative mRNA expression level of the targets genes was normalized for input RNA against *B2M* gene expression in the sample. The relative mRNA expression level (CR) of* sstr2, sstr3, sstr5*, and* Slc6a2 *in each sample was calculated using the comparative cycle time (Ct) method as CR = 2 − (Ct tissue of normal rat − Ct *B2M*) − (Ct target tissue − Ct *B2M*). The average level of the target genes mRNA in tissues of wt animals (+/+) is arbitrarily set to 1. 

### 2.6. Immunohistochemistry

Immunohistochemistry was performed on an automated immunostainer (Ventana Medical systems, Illkirch, France) according to the manufacturer's protocols with minor modifications, as described elsewhere [10]. We used a monoclonal anti-p27Kip1 antibody (BD Transduction Laboratories, Erembodegem, Belgium) and the monoclonal MIB5 antibody (Dako, Glostrup, Denmark) to detect the proliferation antigen Ki67, diluted 1 : 1000 and 1 : 500, respectively. Positive controls (tonsils) were included in each staining run to confirm the adequacy of the staining. Only distinct nuclear staining of tumor cells was used for scoring the Ki67 immunoreactivity, which was determined by counting at least 300 cells each case at high power (400 folds) by a pathologist (A.P.). The number of Ki67 positive cells per 100 adrenal medullary cells is indicated as proliferative index. In the cases in which the number of positive cells was not homogeneous in the adrenal tissue, areas with the lowest and with the highest number of positive cells were selected for counting (Table 1). Images were recorded using a Hitachi camera HW/C20 installed in a Zeiss Axioplan microscope with Intellicam software (Zeiss MicroImaging, Jena, Germany). Adobe PhotoShop (San Jose, CA, USA) was used for image processing. 

### 2.7. Statistical Analysis

Comparisons between standardized uptake values (SUVs) or adrenal to liver ratios measurements in affected versus normal adrenal glands were carried out using the two-sided Student's *t*-test. 

## 3. Results

### 3.1. Animals

**Figure 1:**
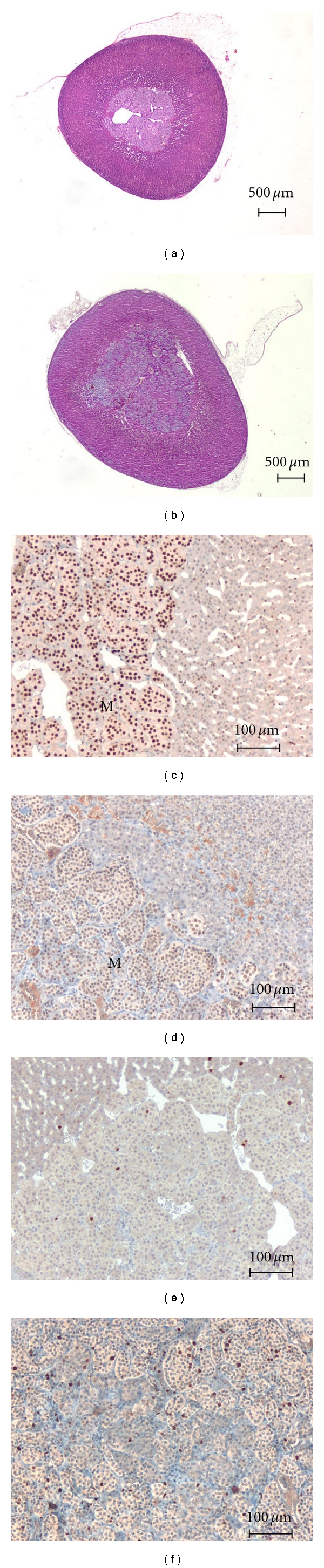
Histopathological and immunohistopathological comparison of MENX mutant rats ((a), (c), (e)) versus normal control rat ((b), (d), (f)). (a) Histology of the right adrenal gland of the normal rat N2 (H&E). (b) Histology of the left adrenal gland of the mutant rat M2 (H&E). (c) Immunohistochemical staining for p27Kip1 of the same gland as in (a). (d) Immunohistochemical staining for p27Kip1 of the same gland as in (b). (e) Immunohistochemical staining for the proliferation marker Ki67 of the same gland as in a. (f) Immunohistochemical staining for the proliferation marker Ki67 of the same gland as in (b). M, adrenal medulla. Original magnifications: (a), (b) ×20; (c)–(f) ×160.

Four mutant animals and three normal controls were subjected to complete analysis with the tracers (^68^Ga-DOTATOC and ^11^C-HED) (Table 1). All adrenal lesions observed in our rats were confirmed by histopathological examination as being pheochromocytomas (Figure 1). The two adrenal glands of each animal were analyzed separately. MENX adrenals were generally larger (237 mm^3^  ±  172) than normal adrenals (107 mm^3^  ±  13). 

### 3.2. PET

**Figure 2:**
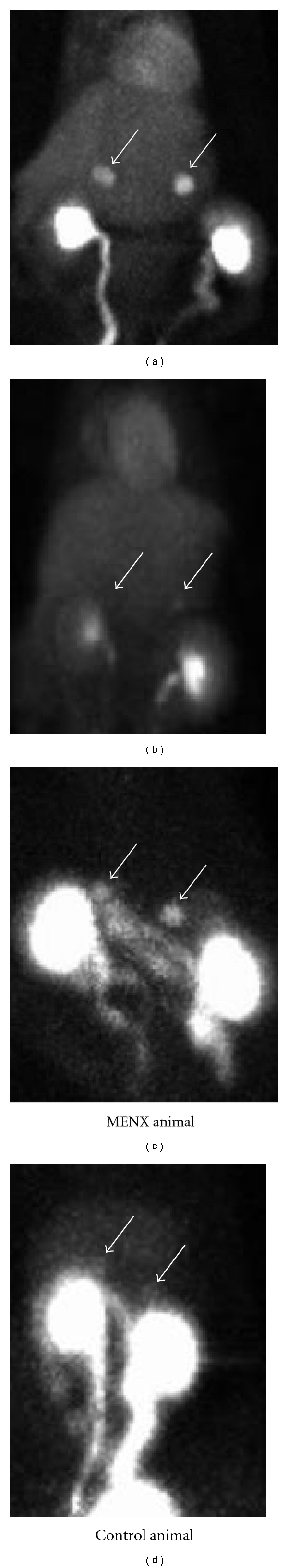
Imaging of rat adrenal glands. (a) ^11^C-HED in a MENX animal. (b) ^11^C-HED in a normal animal. (c) ^68^Ga-DOTATOC in a MENX animal. (d) ^68^Ga-DOTATOC in a normal animal. The arrows indicate the adrenal glands.

**Figure 3:**
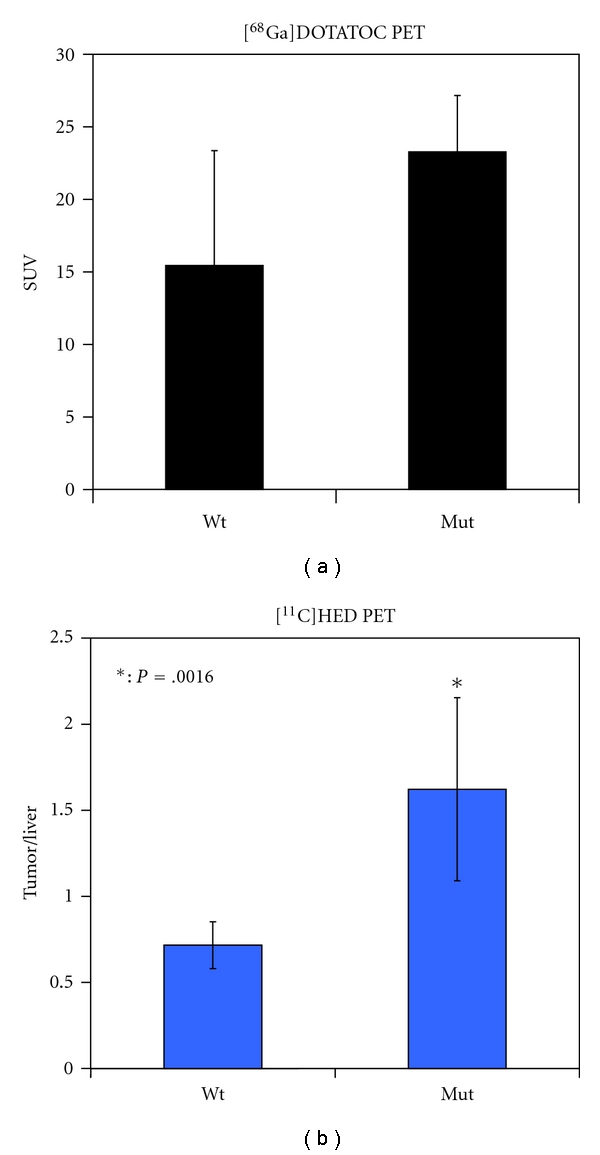
Summary of the uptake values. Uptake values were obtained with the two indicated tracers in normal (Wt) and in mutant rats (Mut). *, *P* = .0016.

Adrenal DOTATOC and ^11^C-HED uptake was detected in all animals (Figure 2). Comparisons between the two groups of animals showed elevated uptake of ^11^C-HED in all mutant rats compared to the controls: the adrenal to liver ratios in mutant rats was 1.6 (±0.5) but was only 0.7 (±0.1) in normal rats (*P* = .002) (Table 1). Also ^68^Ga-DOTATOC uptake was elevated in the adrenal glands of mutant rats compared to the normal controls, although to a lesser extent compared to ^11^C-HED uptake. Indeed, SUV values for ^68^Ga-DOTATOC in pheochromocytoma were 23.3 (±3.9) while were 15.4 (±7.9) in normal control adrenals (*P* = .03) (Figure 3 and Table 1). 

### 3.3. Immunohistochemical Staining for p27Kip1 and Ki67

Affected rats have a germline mutation in *Cdkn1b* that makes the encoded protein highly unstable [11] and as a consequence they have extremely reduced levels, or complete loss, of p27Kip1 in their tissues, as previously reported [9]. We performed immunohistochemical staining of the adrenal glands of the 4 affected and the 3 normal control animals subjected to imaging using an antibody specific for p27Kip1. The results confirmed the low level of p27Kip1 expression in the adrenal medullary cells of the mutant rats compared to those of wild-type control rats (Figure 1). In humans, several normal and neoplastic tissues show an inverse relationship between p27Kip1 expression and the proliferation activity of the tumor cells [12, 13]. Moreover, in human pheochromocytoma the proliferation index is among the parameters that help defining the malignant potential of the tumor [14, 15]. Therefore, we determined the proliferative index of the rat tumor cells by immunohistochemical staining for the proliferation marker Ki67 (MIB-5 antigen) to determine whether the low p27Kip1 levels had an effect on cell proliferation. We found that the average proliferative index in all tumors, except one, varied between 3.7% and 16.7% (Table 1). The exception was the left adrenal tumor of rat M3 that showed a percentage of Ki67 immunopositive cells over 50% (Table 1), which is suggestive of a malignant phenotype. The distribution of Ki67 immunoreactivity in the tumor tissue was not homogeneous, with areas of lower and others of higher proliferative activity. The proliferation rate of all tumors was higher than that of normal rat adrenal glands (Ki67 immunoreactivity <1%).

### 3.4. Real-Time Quantitative RT-PCR for Somatostatin Receptor Genes and Noradrenalin Transporter Gene

**Figure 4:**
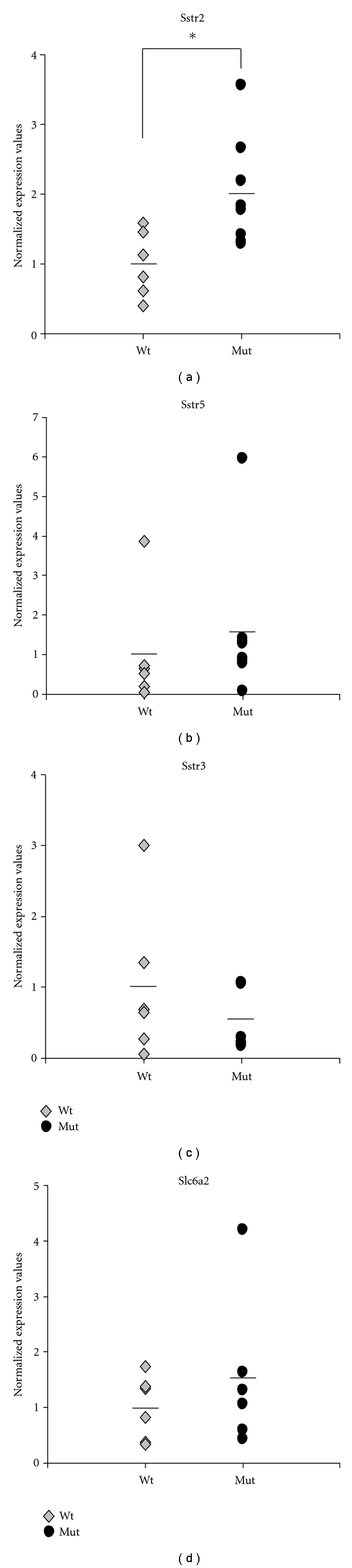
Expression of relevant genes in adrenal glands. Real-time TaqMan RT-PCR of mRNA extracted from normal rat adrenal tissue (wt) and rat tumor tissue (Mut). The ΔCt method was used to normalize gene expression values to the ß2-microglobulin housekeeping control gene and to a reference calibrator RNA. Dots represent individual RNA samples. The bar indicates the average gene expression value. The average value for the normal adrenal tissues was arbitrarily set to 1. *, *P* = .0214.

Neuroendocrine tumor cells often express on their membrane somatostatin receptors (Sstr). Therefore, radiolabeled somatostatin analogues are useful in the clinical management of neuroendocrine malignancies and also in their detection by functional imaging. Among the different Sstr subtypes identified (Sstr1-Sstr5), Sstr2 shows the highest affinity for the somatostatin analogues such as ^68^Ga-DOTATOC, followed by Sstr5 and Sstr3 [16]. Recently, standardized uptake values (SUV) with ^68^Ga-DOTATOC-PET in patients with neuroendocrine tumors have been found to correlate with the expression of the SSTR2 protein by the tumor cells [17]. To determine if there might be a correlation between uptake values in MENX rats and expression of the somatostatin receptors, we quantified the level of the rat *Sstr2*, *Sstr3,* and *Sstr5* genes by Real-Time quantitative RT-PCR in the adrenal lesions subjected to functional imaging. Similarly, we also analyzed the expression of the *Slc6a2* gene encoding for the norepinephrine transporter, the protein implicated in the reuptake of monoamines, including ^11^C-HED (the other tracer we used), in chromaffin cells. The results show that only the expression of the *Sstr2 *gene encoding, the somatostatin receptor subtype having the highest affinity for ^68^Ga-DOTATOC, is slightly but significantly increased in affected *versus* control adrenals (2.1-fold ±0.8; *P* = .0214, two-tailed *U* test). In contrast, the mRNA level of the *Sstr3* (80.5 ± 0.4; *P* = .63), *Sstr5* (1.6 fold ±1; *P* = .093), and *Slc6a2* (1.5 fold ±1.4; *P* = .42) genes are not significantly different in affected *versus* wild-type control rats (Figure 4). 

## 4. Discussion

Our study shows that functional imaging can visualize noninvasively the pheochromocytomas that develop in the context of the MENX rat syndrome. These findings set the standard for future preclinical testing of novel targeted therapies for neuroendocrine tumors in this animal model. 

^11^C-HED gave the best imaging results (Table 1) suggesting that the norepinephrine transport system might be a more suitable target to visualize MENX-associated pheochromocytoma by PET-imaging. Quantitative RT-PCR showed that the expression of the *Slc6a2* gene (norepinephrine transporter) is comparable between mutant and wild-type animals. This indicates that the level of expression of *Slc6a2* mRNA does not highly correlate with ^11^C-HED tracer uptake, as affected rats show a much higher uptake of the tracer than control rats despite having an mRNA level similar to the controls. Therefore, in our model system the level of expression of the *Slc6a2* gene is not predictive of the protein amount and/or of the biological activity of the norepinephrine transporter system. Of course we cannot exclude that differences might exist between mutant and control rats in the final amount of norepinephrine transporter protein or in its activity.

Imaging with the somatostatin receptor analogue ^68^Ga-DOTATOC demonstrated a moderate increase in the uptake of this tracer in affected rat adrenal glands compared to wild-type control glands. This is in agreement with the increased expression level of the *Sstr2* gene in mutant compared to normal rat adrenals. However, Ga-68-DOTATOC uptake was lower than we expected in the rat tumor tissues, and quantification of the somatostatin receptor protein in these cells might be required to fully clarify this issue.

Immunohistochemical staining confirmed the low levels of p27Kip1 in the mutant rat adrenals and showed a moderate to elevated proliferation index of the tumors. The proliferation rate of the rat pheochromocytomas was not homogeneous within the lesions and varied between 1.6 and 22.5%, with one adrenal gland (of animal M3) having a proliferation index above 50% (Table 1). Immunoreactivity for Ki67 in the rat lesions was higher than that displayed by human pheochromocytoma, usually lower than 3.5% [14, 18, 19]. In patients a high proliferation rate is most frequently found in malignant tumors [7]. Therefore, the MENX pheochromocytoma with high Ki67 immunoreactivity has characteristics suggestive of an aggressive behaviour. No metastatic spread was observed both by histology and by PET in any of the affected animals subjected to imaging, maybe because it had not yet taken place as the mutant rats were relatively young (5 months old) and their average life-span is 10 ± 2 months. In any case, the relatively high Ki67 proliferation index makes MENX an interesting model of aggressive behaviour in pheochromocytoma.

One of the PET-tracers used in staging and restaging of human pheochromocytoma is the norepinephrine analogue ^11^C-HED (Hydroxyephedrine). Compared to ^123^I-MIBG, a norepinephrine analogue commonly used for conventional scintigraphy including SPECT, ^11^C-HED is a more polar molecule resulting in faster excretion and therefore in the possibility of early, time-saving imaging acquistion [20]. In addition to ^11^C-HED, ^68^Gallium labelled somatostatin analogues (such as ^68^Ga-DOTATOC) are clinically suitable for the imaging of human pheochromocytoma [21]. Both norepinephrine uptake and somatostatin receptor binding have been used as targets for internal delivery of beta emitting isotopes for palliative treatment of metastasized paragangliomas or pheochromocytomas [22]. Further investigation of such targeted radionuclide therapy approaches in the MENX rat model might improve their application in humans. 

For both tracers (^11^C-HED and ^68^Ga-DOTATOC), slightly different methods of quantification of the PET signal were chosen. For ^11^C-HED, liver to tumor ratios were chosen according to clinically established methods for diagnosis of pheochromocytoma using ^131^I-MIBG-scintigraphy [23]. This approach quantifies the norepinephrine transporter system, which also mediates ^11^C-HED internalization. Since ^68^Ga-DOTATOC-PET did not show relevant tracer uptake in the rat liver, it is impossible to delineate this organ on the scans. Therefore, a quantification factor has been established to yield absolute activity concentrations and derived standardized uptake values *in vivo* without the need of a reference region.

Internal radiotherapy of pheochromocytoma with ^131^I-MIBG has been used with some success in reducing tumor bulk, thereby attenuating the symptoms associated to catecholamines oversecretion and alleviating pain. However, a curative potential has generally not been observed. Additionally, cytoreductive chemotherapy has been applied with limited success [24]. In this context, novel compounds for targeted therapy are needed. The ongoing molecular chacterization of MENX-associated pheochromocytoma and precursor lesions will identify new specific pathways involved in tumor development. If suitable, these novel molecular targets will be used for small animal PET imaging, to further characterize the MENX animal model and to evaluate new targeted treatment modalities.

The mutant p27 protein associated with the MENX syndrome is degraded very fast by the proteasome [11] so that its level in the tissues of affected rats is extremely reduced or totally absent [9]. In humans, the expression level of p27 is also frequently reduced or absent in many tumor types [25], and this downregulation is achieved mainly through accelerated proteasome-mediated proteolysis [26], establishing a parallel with the situation in MENX rat tumors. Due to the tumor suppressive function of p27, restoring its levels in tumor cells may reinstate control of cell proliferation, hence the interest in compounds that might specifically interfere with p27 degradation [27]. MENX-affected animals represent an ideal preclinical model for investigating the effect of novel proteasome-inhibitor drugs on pheochromocytoma. Based on our findings, monitoring of the efficacy of such compounds against the tumor can be achieved by performing repeated ^11^C-HED-PET imaging.

In conclusion, the MENX rat model, that develops pheochromocytoma with 100% penetrance, in conjunction with the imaging approaches described here, might be a powerful preclinical model system to evaluate different targeted therapy strategies like, for example, targeted radionuclide therapies or novel proteasome inhibitors in pheochromocytoma. Tracer uptake was able to distinguish affected from normal adrenal glands, indicating that the resolution of the method is appropriate for the precise localization of the rat adrenal tumors. This high resolution in combination with specific PET tracers will allow us to precisely monitor the dynamics of tumor development following treatment of young affected rats with therapeutic compounds with antitumor activity. Further studies will extend the range of suitable PET-tracers that depict distinct pathways of pheochromocytoma tumorigenesis, such as tracers investigating additional cell surface markers (such as integrins or other neuroendocrine-specific receptors) or metabolic pathways (e.g., ^18^F-DOPA). 

##  Conflict of Interests

The authors declare that they have no conflict of interests. 

## Figures and Tables

**Table 1:** Anatomical and functional quantification of the adrenals in MENX mutant rats compared to normal controls.

	Right adrenal				Left adrenal			
	[^68^Ga]DOTATOC SUV	[^11^C]HED adrenal/liver	Volume (mm^3^)	Proliferation index (range)	[^68^Ga]DOTATOC SUV	[^11^C]HED adrenal/liver	Volume (mm^3^)	Proliferation index (range)

Wild-type control rats (wt/wt)								

N1	23.189	0.921	120.7	<2%	25.331	0.780	96.3	<2%
N2	15.177	0.725	98.35	<2%	15.703	0.720	91.62	<2%
N3	6.944	0.634	121.0	<2%	6.301	0.517	116.86	<2%

Mutant rats (mut/mut)								

M1	21.479	1.370	162.24	6.6% (4.2–10.2)	24.077	1.326	168.94	16.7% (4.2–12.5)*
M2	19.088	1.270	165.51	3.7% (1.6–5.7)	16.857	1.368	176.99	11.6% (4.3–18.9)*
M3	23.797	2.193	638.93	13% (3.4–22.5)*	26.096	2.709	317.11	52% (50–54)
M4	28.173	1.361	132.67	n.d.	26.598	1.375	135.71	5.1% (2.8–8.3)

*Ki67 staining pattern is very heterogeneous: some areas have low, others have high Ki67 immunoreactivity.
